# Source Apportionment of Atmospheric PM_10_ in Makkah Saudi Arabia by Modelling Its Ion and Trace Element Contents with Positive Matrix Factorization and Generalised Additive Model

**DOI:** 10.3390/toxics10030119

**Published:** 2022-03-02

**Authors:** Turki M. Habeebullah, Said Munir, Jahan Zeb, Essam A. Morsy

**Affiliations:** 1 Department of Environmental and Health Research, The Custodian of the Holy Two Mosques Institute for Hajj and Umrah Research, Umm Al Qura University, Makkah 24382, Saudi Arabia; tmhabeebullah@uqu.edu.sa (T.M.H.); jzhabib@uqu.edu.sa (J.Z.); eamibrahim@uqu.edu.sa (E.A.M.); 2 Faculty of Environment, Institute for Transport Studies, University of Leeds, Leeds LS2 9JT, UK

**Keywords:** PM_10_, source apportionment, ions, trace elements, PMF, GAM, air pollution, Makkah

## Abstract

In this paper, the emission sources of PM_10_ are characterised by analysing its trace elements (TE) and ions contents. PM_10_ samples were collected for a year (2019–2020) at five sites and analysed. PM_10_ speciated data were analysed using graphical visualization, correlation analysis, generalised additive model (GAM), and positive matrix factorization (PMF). Annual average PM_10_ concentrations (µg/m^3^) were 304.68 ± 155.56 at Aziziyah, 219.59 ± 87.29 at Misfalah, 173.90 ± 103.08 at Abdeyah, 168.81 ± 82.50 at Askan, and 157.60 ± 80.10 at Sanaiyah in Makkah, which exceeded WHO (15 µg/m^3^), USEPA (50 µg/m^3^), and the Saudi Arabia national (80 µg/m^3^) annual air quality standards. A GAM model was developed using PM_10_ as a response and ions and TEs as predictors. Among the predictors Mg, Ca, Cr, Al, and Pb were highly significant (*p* < 0.01), Se, Cl, and NO_2_ were significant (*p* < 0.05), and PO_4_ and SO_4_ were significant (*p* < 0.1). The model showed R-squared (adj) 0.85 and deviance explained 88.1%. PMF identified four main emission sources of PM_10_ in Makkah: (1) Road traffic emissions (explained 51% variance); (2) Industrial emissions and mineral dust (explained 27.5% variance); (3) Restaurant and dwelling emissions (explained 13.6% variance); and (4) Fossil fuel combustion (explained 7.9% variance).

## 1. Introduction

Air pollution is one of the major public health concerns causing cardiovascular, pulmonary, respiratory, and cognitive problems. PM_10_ (particulate matter of aerodynamic diameter up to 10 μm) has been associated with millions of premature mortalities worldwide [1]. Air pollution is the cost of the rapid growth of urbanisation and industrialisation, and as a result, the air quality in large urban areas is getting worse day by day. Urban air quality has caught the interest of researchers worldwide, analysing emission sources, levels of various air pollutants, and predicting future concentrations and their impacts on human health [2,3,4,5]. In addition to the natural sources of air pollutants (e.g., dust storms, volcano eruptions, and forest fires), anthropogenic sources contribute a significant proportion of the pollutant loads to the atmosphere [6,7]. Therefore, numerous investigations have been made to quantify the contribution of each emission source (e.g., [8,9]).

Air pollution levels in the atmosphere are controlled by emission sources, meteorological conditions, and geographical characteristics. Previously, several studies have reported that particulate pollution is a serious environmental issue in Middle Eastern countries, including Saudi Arabia [10,11,12,13,14,15,16,17], which in addition to arid geographical conditions and frequent sand storms, is encouraged by rapid urbanisation and industrialisation. Saudi Arabia has large scale desert areas, arid or semi-arid climatic conditions, little rain, and strong winds, which play a positive role in enhancing the atmospheric burden of particulates. These natural conditions are supported by anthropogenic activities including busy roads, large and small scales industries, construction-and-demolition projects, and oil and coal burnings [11,18,19].

Air quality in Makkah has been analysed by several researchers in the past. Habeebullah et al. [20] carried out source apportionment of PM_10_ based on ionic analysis. In the study, SO_4_^2−^, NO_3_^−^, Ca^2+^, Na^+^, and Cl^−^ were the major contributors, contributing 37.81% to PM_10_ concentrations. Road traffic, mineral dust, industries, construction and demolition, sea spray, and marine aerosols were identified as the major emission sources of PM_10_ [20]. Farahat et al. [10] monitored air quality in Makkah, Madinah, and Jeddah during the Hajj seasons of 2019 and 2020 and compared the levels in the two years. They concluded that concentrations of PM_10_, NO_2_^−^_,_ and CO were reduced during the Hajj seasons in Makkah in 2020 compared to 2019 from 27.45 to 34.65 µg/m^3^, 9.95 to 4.50 ppbv, and 1.28 to 0.81 ppm, respectively. During the Hajj 2019 season, there were no restrictions, whereas in 2020, due to COVID-19, the number of pilgrims was restricted, therefore, the reductions seemed to be caused by the COVID-19 lockdown restriction [10]. Nayebare et al. [8] analysed PM_2.5_ samples to determine their emission sources in Makkah for a year. Average 24 h PM _2.5_ concentration (35 µg/m^3^) was observed to be higher than the WHO standard (25 µg/m^3^). They reported that the Air Quality Index (AQI) was “unhealthy to hazardous.” According to this study, vehicular emission, industrial mixed dust, earth crust, and fossil fuel combustion were determined to be the major sources of PM_2.5_ [8].

The focus of this study is to measure the concentrations of several ions (Na^+^, Ca^2+^, Mg^2+^, F^−^, Cl^−^, Br^−^, NO_2_^−^, NO_3_^−^, SO_4_^2−^, and PO_4_^3−^) and trace elements (Pb, As, Cd, Cr, Se, and Al) in PM_10_. This study builds on Habeebullah et al. [20], which analysed the concentrations of water-soluble ions with Principal Component Analysis (PCA) to identify the potential sources of PM_10_. In this study, the range of chemical species is increased, and advanced modelling approaches are employed, namely Generalised Additive Model (GAM), Positive Matrix Factorisation (PMF), and Enrichment Factor (EF), along with graphical visualization and correlation analysis to identify the main emission sources of PM_10_ and investigate the linear and nonlinear association between the various constituents of PM_10_.

## 2. Materials and Methods

### 2.1. Description of the Monitoring Sites

Makkah is the Holy City for all Muslims around the world. Makkah is situated in the western part of Saudi Arabia, among the Sarawat Mountains, about 80 km inward from the Red sea, and 277 m above sea level [10]. The population of Makkah city is around 1.96 million, and the population of the Makkah region is more than 8.5 million, with a growth rate of 1.8% [21,22]. Makkah is unique in the sense that it receives an additional 3 to 4 million pilgrims during the month of pilgrimage (Hajj) and Ramadhan (fasting) every year, except in 2020 and 2021, when Hajj was cancelled due to the COVID-19 pandemic [23]. The vast number of pilgrims leads to traffic congestion within and around the city, resulting in high levels of exhaust and non-exhaust emissions and dust resuspension [24]. Makkah experiences hot and dry climatic conditions, where the maximum temperature reaches 55 °C in the summer season [25].

**Figure 1:**
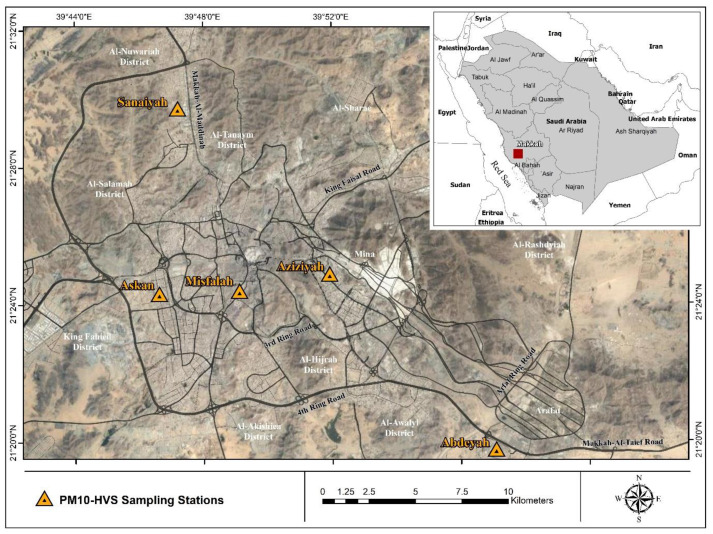
Location map of the five sampling sites (Aziziyah, Misfalah, Sanaiyah, Abdeyah, and Askan) in Makkah [20].

The data analysed in this paper were collected at five sites around the city, namely Aziziyah, Sanaiyah, Abdeyah, Misfalah, and Askan (Figure 1). Aziziyah and Misfalah are considered urban traffic sites, Sanaiyah an industrial site, Askan a residential site, and Abdeyah a background site. The major emission sources in Makkah are related to road traffic, including both exhaust and non-exhaust emissions and the resuspension of dust particles on roadsides [20]. Makkah is not an industrial city like Jeddah; however, there is a power plant in Sanaiyah. Furthermore, emissions from restaurants and dwellings need to be quantified.

### 2.2. Collection of PM_10_ Samples

High-volume samplers were used to collect PM_10_ samples from 8 March 2020 to 9 March 2021 at the five sites in Makkah. These samplers used high–volume glass fibre filters (8 × 10 inches, grade G 653, Whatman, Maidstone, UK) with inlet collection efficiency of a cut–point of 9.7 microns and flow rate of 1.13 m^3^/min. Before deploying in the field, the filters were put in an oven (LDO–060E, Lab Tech, Daejeon, Korea) at 300 °C for 5 h to remove their moisture and organic contents [26,27,28,29]. Samples were collected for 24-h periods covering all days of the week. The filters of the samples were collected strictly from 9:00 to 10:00 am. Samples were not collected from 7 April 2020 to 31 May 2020 due to the COVID-19 lockdown in Makkah. After collection, the samples were transported to the laboratory at the Custodian of the Two Holy Mosques Institute for Hajj and Umrah Research, Umm Al-Qura University Makkah, where the filters were kept at room temperature for 24 h in a desiccator. An analytical balance was used to check the weight of the filters until a constant weight was obtained, which was considered the final weight of the filters. The filters were then stored in the laboratory, while sealed in a polythene bag until the time of analysis [26,27,28,29].

### 2.3. Analysis of Water-Soluble Ions and Metal Contents

Before analysis, the filters were weighed again. Then each filter was cut into four equal portions and stored at 4 °C in a refrigerator. Each piece was analysed for different constituents. PM_10_ concentrations were calculated using the mass of the collected samples on the filters (the difference in the mass of the filters before and after exposure in the air for 24 h), and the volume of the air passed through the filters [26].

The filters were analysed for various ions in the PM_10_ samples. One-quarter of the filter was analysed for water-soluble ions, namely fluoride (F^−^), chloride (Cl^−^), nitrite (NO_2_^−^), bromide (Br^−^), nitrate (NO_3_^−^), sulphate (SO_4_^2−^), phosphate (PO_4_^3−^), sodium (Na^+^), calcium (Ca^2+^), and magnesium (Mg^2+^). For this purpose, the filter was shredded into 25 mL deionised distilled water with a resistivity of 18 Ωcm in a conical flask. The conical flask was ultrasonicated for 1 h in an ultrasonic bath (ATM40–28LCD, Ovan, Badalona, Spain). To remove undissolved particles, the solution was filtered through a 0.45 μm pore size membrane (CHROMAFIL, CA–45/25 (S), Macherey-Nagel, Düren, Germany) and stored in a refrigerator at 4 °C [30]. Ion Chromatography (850 Professional, Metrohm, Herisau, Switzerland) was used to determine the ion concentrations in the extract. Then, HNO_3_ (3.2 mM) and Na_2_CO_3_ (1.8 mM) were used to dissolve cations and anions, respectively, using a flow rate of 0.7 mL/min and injection volume of 10 µL [31]. For quality assurance, triplicate samples and blank calibrations were carried out. The detection limit (ppm) of the ion chromatography was 0.001 for fluoride and phosphate, 0.002 for sodium and magnesium, and 0.005 for chloride, nitrite, bromide, nitrate, and calcium.

For metal analysis, a portion of the filter paper was digested using the ‘hot acid extraction procedure’ (US EPA Method IO-3.1). The filter paper was shredded into strips in a 150 mL small flask, adding 10 mL of freshly prepared regal water (HCl:HNO_3_, 75 mL:25 mL). Filter strips were made to be set down in such a way that they were covered by the acid mixture entirely. The flask was placed on the hot plate at 60–70 °C for 60 min and was not allowed to dry. Once digestion was completed, the flask was removed from the hot plate and allowed to cool. The flask walls were rinsed with 20 mL deionized water and left for another 30 min, allowing acid to diffuse into the water from the sample filter. A PTFE syringe filter (Lichen Cottage Syringe filters, Marlborough, MA, USA) 0.45 µm in size was used to filter the digested material into a 50 mL volumetric flask and filled up to the mark [32].

The concentrations of heavy metals were determined with a graphite furnace using an atomic absorption spectrophotometer, Thermo Scientific (ice 3000 series, Waltham, MA, USA). A deuterium lamp was used by the instrument to make background corrections for every reading. Stock calibration standards for each analysed metal (Pb, As, Cd, Cr, Se, Al) were made by 1000 ppm (Sigma-Aldrich, Saint Louis, MO, USA) standard for respective metals. Calibration standards for each metal were made based on concentration to absorbance values.

For QA procedures, respective certified reference materials (CRM) were used the same way as the samples. NIST 1648a (National Institute of Standards and Technology, Gaithersburg, MD, USA), IC-6-1 (nsi lab solutions, Raleigh, NC, USA), and IC-7-2 (nsi lab solutions, USA) were used as the CRM for metals, cations, and anions, respectively. Recoveries show satisfying results for the analyzed concentrations [33,34].

### 2.4. General Statistical Analysis

The speciated data of PM_10_ was analysed using R programming language [35] and three of its packages: ‘openair’ (version 2.8-6) [36], ‘mgcv’ (version 1.8-39) [37], and ‘ggplot2’ (version 3.3.5) [38]. Correlation analysis was performed to investigate the linear relationship between different ions. A correlation plot was developed in the openair–package [36] using its function ‘corPlot’ (version 0.92). The ‘mgcv-package’ was used for the implementation of GAM, and PMF (version 5.0) was used to identify the main emission sources of PM_10_ in Makkah.

### 2.5. Positive Matrix Factorization

Positive matrix factorization (PMF) is one of the most widely employed models for the source apportionment of different air pollutants [39]. PMF (version 5.0) was employed for the source apportionment analysis to identify the major sources of PM_10_ in Makkah, which explained over 80% variation in PM_10_ concentrations. PMF has proven to be a powerful technique for particulate matter source apportionment, which was developed by [40]. For more details on PMF and receptor modelling, the readers are referred to [41]. PMF solves the chemical mass balance equation between measured pollutant concentrations and source profiles as presented in Equation (1) [42]:

(1) $$X_{\mathrm{ij}}=\sum_{k=1}^{p}g_{ik}f_{kj}+e_{ij}$$

In Equation (1), ‘*p*’ is the number of factors, ‘*f*’ is the species profile of each source, ‘*g*’ is the amount of mass contributed by each factor to each individual sample, and ‘*e*’ is the error (residual) of each species.

### 2.6. Generalised Additive Model (GAM)

GAM is a type of supervised machine learning model initially developed by [43], which combines the properties of generalized linear models with additive models. GAM is an advanced nonlinear model, which does not assume linearity between the response and explanatory variables and does not require the modelled variable to be normally distributed, permitting the response probability distribution to be any member of the exponential family (e.g., normal, exponential, gamma, Poisson and many other) [44]. For a given response variable, Y regressed over ‘m’ explanatory variables X_1_, X_2_, ..., X_m_, a GAM in a general form can be described as shown in Equation (2):

Y = s_1_ (X_1_) + s_2_ (X_2_) + … + s_m_ (X_m_) (2)

where Y is the response (PM_10_ concentrations in Equation (3)) variable and ‘s’ is the smoothing term, which corresponds to an associated explanatory (independent or predictor) variable (X). Using the above general form, the model developed in this study can be presented as below (Equation (3)):

PM_10_ ~ s1 (Pb) + s2 (Cd) + s3 (Cr) + s4 (As) + s5 (Se) + s6 (Al) + s7 (F) + s8 (Cl) + s9 (NO_2_) + s10 (Br) + s11 (NO_3_) + s12 (PO_4_) + s13 (SO_4_) + s14 (Na) + s15 (Ca) + s16 (Mg) (3)

### 2.7. Enrichment Factor

Enrichment factor (EF) has been widely used to calculate the extent of anthropogenic contribution in comparison to the earth crust. The main aim of EF is to determine the sources of particulate matter, here PM_10_. Generally, aluminium (Al) is used as a reference element due to its abundance in earth crust. Several researchers (e.g., [8,9,45,46]) have previously used EF to identify the two main sources (anthropogenic sources and crustal earth) of trace elements in PM_10_ and PM_2.5_.

To calculate EF the following formula was used:

Ratio 1 = Concentration of TE in PM_10_/Concentration of Al in PM_10_ (4)

Ratio 2 = Concentration of TE in earth-curst/Concentration of Al in earth crust(5)

EF = Ratio 1/Ratio 2(6)

In Equations (4)–(6), EF stands for enrichment factor, TE for trace elements, whose emission sources are to be determined, and Al (aluminium) is the reference element. The concentrations of TE and Al in PM_10_ were determined as a part of this project, whereas the concentrations of TE and Al in earth crust were taken from [47]. An EF value of a TE less than 10 shows that a significant proportion of the TE is emitted from the earth’s crust, whereas an EF value of greater than 10 shows that the TE is mainly emitted by anthropogenic sources [8,9].

## 3. Results and Discussion

### 3.1. Descriptive Analysis of PM_10_ and Its Constituents

**Figure 2:**
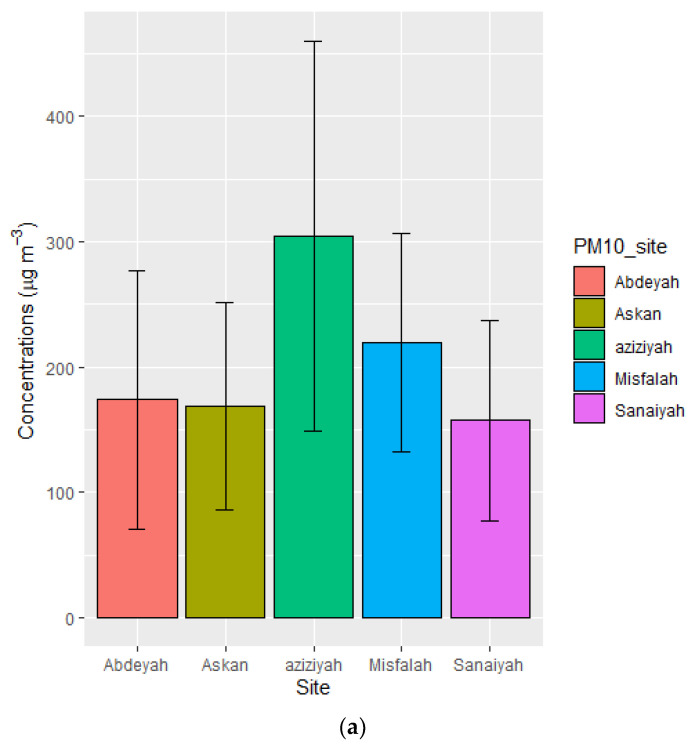

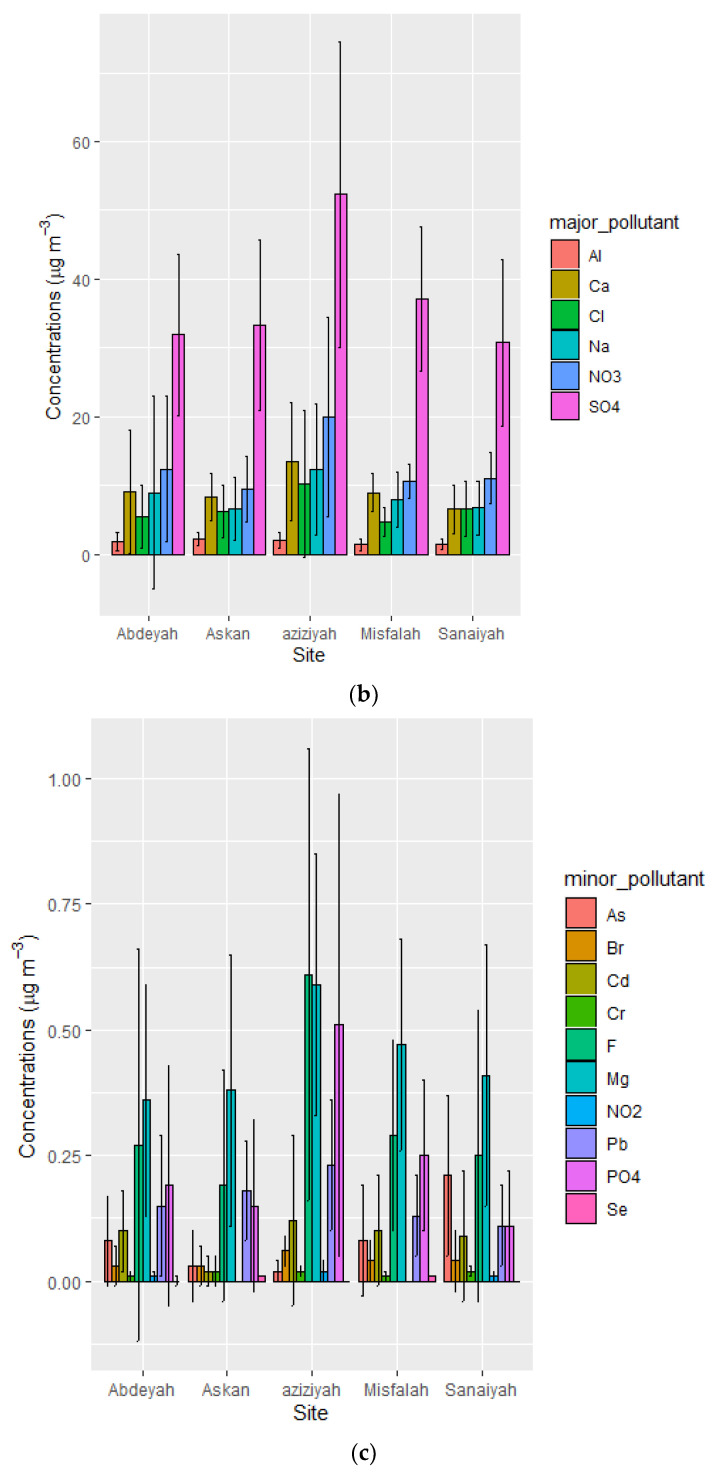
The concentrations of PM_10_ (**a**), major constituents (**b**), and minor constituents (**c**) at different monitoring sites in Makkah.

**Figure 3:**
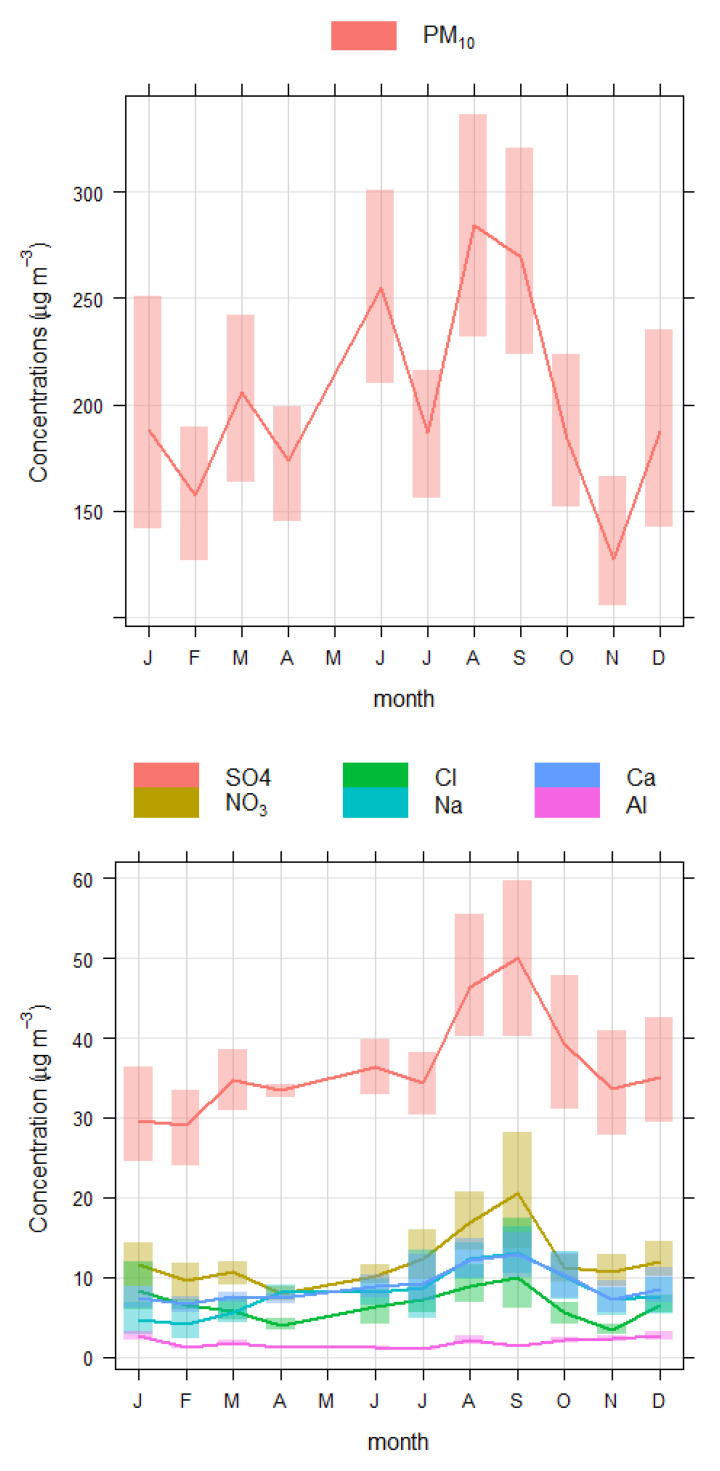

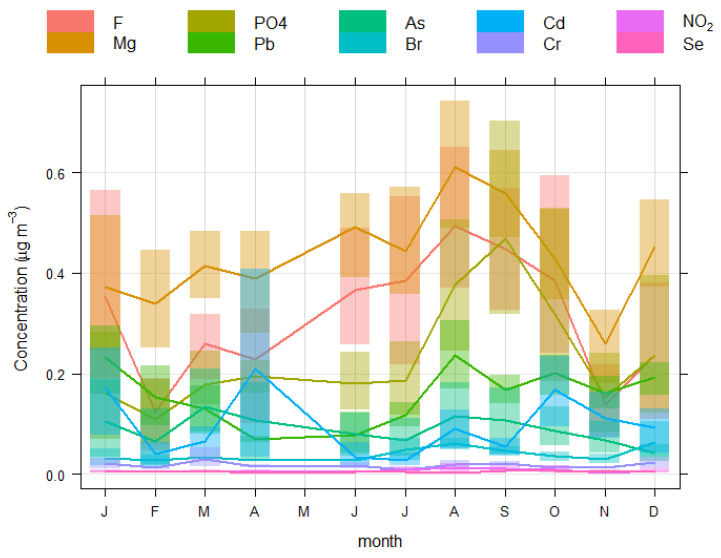
Time variations of monthly average PM_10_(**upper-panel**), major constituents (**middle-panel**), and minor constituents (**lower-panel**) during the study period.

The levels of PM_10_ (µg/m^3^) at different monitoring sites are depicted in Figure 2, which showed that annual average PM_10_ concentrations exceeded the Saudi Arabia national annual air quality standards of 80 µg/m^3^ [48]. Annual average air quality standards developed by WHO (15 µg/m^3^) and USEPA (50 µg/m^3^) are even lower [49]; therefore, the WHO and EPA standards were exceeded as well. The annual average of PM_10_ concentrations (µg/m^3^) were 304.68 ± 155.56 at Aziziyah, 219.59 ± 87.29 at Misfalah, 173.90 ± 103.08 at Abdeyah, 168.81 ± 82.50 at Askan, and 157.60 ± 80.10 at Sanaiyah, in descending order. Average concentrations of PM_10_ and its major and minor constituents are depicted in Figure 2, and the time series of the monthly average is depicted in Figure 3. Aziziyah experienced the highest concentrations of PM_10_, ion contents, and trace elements, followed by the Misfalah site. These two sites are among the busiest urban areas in Makkah and remain very busy during the month of Hajj (Pilgrimage) and Ramadhan (fasting). However, Makkah also remains busy during other times of the year. It should be noted that in 2020 Hajj was cancelled due to the COVID-19 pandemic lockdown. Figure 3 showed that the levels of PM_10_ and its constituents were higher in summer (June to September), which could be due to seasonal effects, as generally, summer experiences the most number of dust storms in Saudi Arabia [19]. Missing data in May was due to the COVID-19 pandemic lockdown in Makkah.

High PM_10_ levels may pose a serious risk to the health of residents and visitors. Previously, several authors have reported high levels of particulate matter in Makkah e.g., [23]. Several reasons have been reported for the high levels of particulate matter in Makkah and the surrounding areas. Farahat [50] reported that large-scale infrastructure activities, overusing governmental subsidized energy, water desalination, heavy traffic in urban areas, and cement plants were the main reasons for the high levels of pollutants. Saudi Arabia is situated within a semi-arid region, and a major part of the country is occupied by deserts. Strong wind in the summer season uplifts desert soil and sand into the atmosphere, which causes heavy aerosol loading [18]. Due to large desert areas, frequent sandstorms, and little rainfall, the desert areas and the urban areas of Saudi Arabia experience higher concentrations of particulate matter [9,50,51].

### 3.2. Correlation Analysis

Correlation analysis shows the linear relationship between two variables. The strength of the relationship is expressed in terms of correlation coefficients (r), which range from −1 to +1, where zero shows no linear relationship, and ±1 shows a hundred percent positive/negative linear relationship.

**Figure 4:**
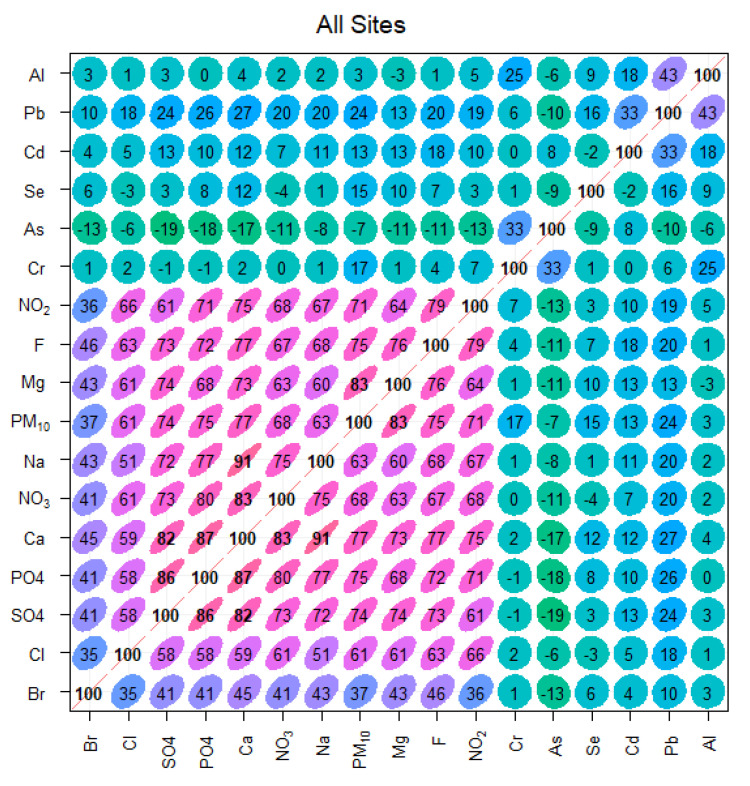
Correlation plots of PM_10_, trace elements, and ions for ‘all sites’ in Makkah (individual sites are not shown for brevity).

The R-values between PM_10_ and its major and minor constituents are presented in the shape of correlation plots in Figure 4. The strength of the R-values is defined by the shape of the circle, its colour, and the number written inside. The R-values between the different chemical species varied from site to site (Figure 4). To provide an overall picture of the correlation, we combined the data from all five sites and calculated the R-values (Figure 4). Combining the data from all sites, we increased the power of the statistical test by increasing the number of observations of each chemical species [8]. PM_10_ showed the strongest correlation with Mg (0.83), followed by Ca (0.77), and the weakest correlation with Al (0.03), followed by As (−0.07). The strongest correlation was found between Na and Ca (0.91), followed by Ca and PO_4_ (0.87), considering all chemical species. Most of the pollutants had a positive association with each other, except As, which was negatively associated with all pollutants, except Cd and Cr (Figure 4). However, the correlation varied from site to site (not shown for brevity), probably indicating variations in their emission sources and factors responsible for their dispersion, dependent on the local micro-level environmental and infrastructure conditions. Generally, if two chemical species are emitted by the same source, they will have a high positive correlation coefficient. Otherwise, they will show a weak or even negative correlation coefficient. Furthermore, those pollutants emitted by the dominant sources that strongly affect PM_10_ concentrations (e.g., road traffic), will have stronger correlation coefficients with PM_10_. Ion concentrations dominate the PM_10_ concentrations and have a stronger effect on PM_10_. Therefore, they have stronger correlation coefficients than metals. However, linear correlation simply shows a relationship between a pair of pollutants and does not take into account the interaction between various pollutants. In addition, the relationship between different pollutants is not always linear (as shown in Section 3.3). Therefore, further advanced analysis is provided in the coming sections.

### 3.3. Generalised Additive Model (GAM)

As discussed in Section 3.2, the strength of the correlation coefficient is based on the linear association between two chemical species. However, the relationship between the two species is not always linear. Therefore, in this section, a nonlinear machine learning approach was employed to provide further insight into the association between PM_10_ and its constituents and see how much variation in PM_10_ concentrations could be explained by the predictors analysed here.

**Figure 5:**
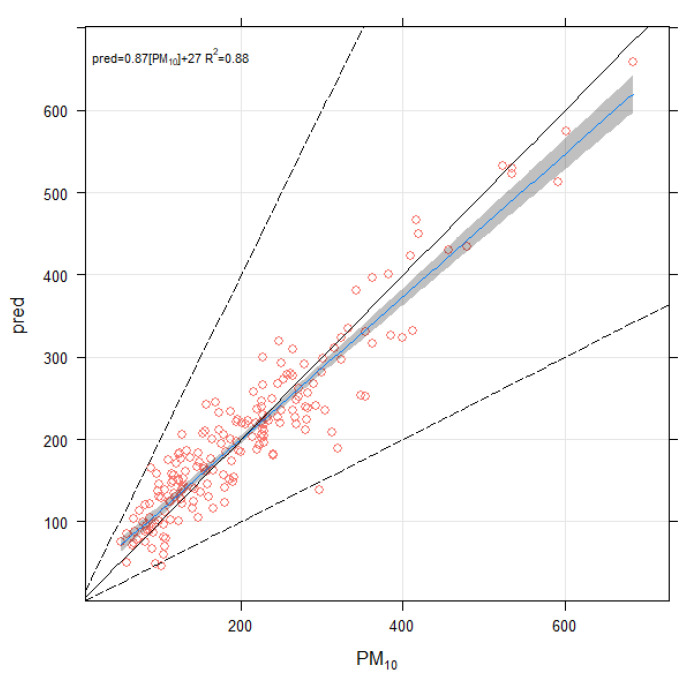
Scatter plot between measured and GAM predicted PM_10_ concentrations (µg/m^3^). The 1:1 line is solid black and the 1:0.5 and 1:2 lines are dashed black. Together these lines help show how close a group of points are to a 1:1 relationship and show the points that are within a factor of two (FAC2). The solid blue line shows the linear model line with 95% confidence intervals. The equation of the line and R-square value is shown in the top-left of the panel.

PM_10_ was used as the modelled (response or dependent) variable, and all constituents (TEs and ions) were used as predictor (explanatory or independent) variables. Among the predictors, Mg, Ca, Cr, Al, and Pb were highly significant (*p*-value < 0.01, ‘**’), Se, Cl, and NO_2_ were significant (*p* < 0.05, ‘*’), and PO_4_ and SO_4_ were significant (*p* < 0.1, ‘.’). The model showed an adjusted r-squared of 0.85, and deviance explained 88.1%. Deviance is a measure of goodness-of-fit of a model, similar to the R^2^ of Gaussian data. Several other statistical metrics were calculated by comparing observed and predicted PM_10_ concentrations, including correlation coefficients (0.94), a factor of two (0.99), mean biased error (1.19e^−9^), and root mean squared error (8.89 µg/m^3^). Definitions and formulae for calculating these statistical metrics can be found in [52,53]. Predicted and observed PM_10_ concentrations are compared in Figure 5 in the form of a scatter plot, which showed a strong association between predicted and measured concentrations of PM_10_.

**Figure 6:**
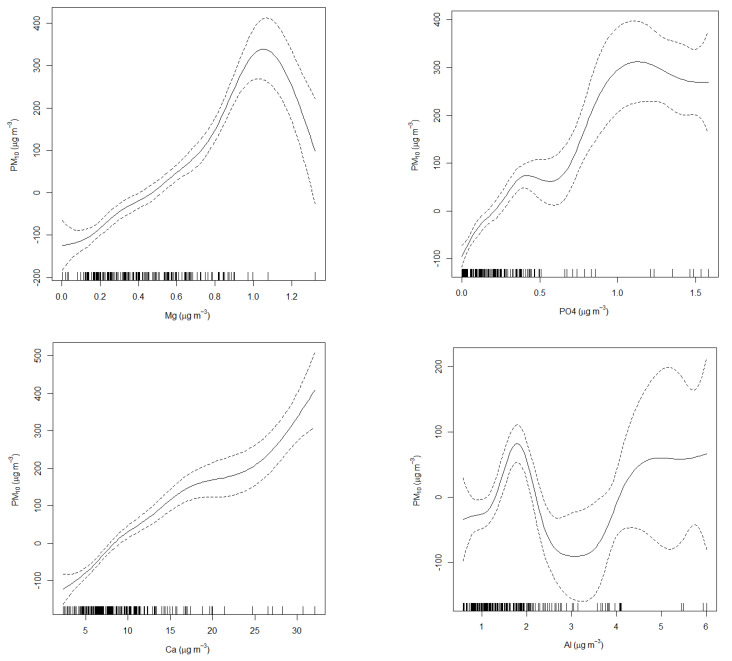
Showing the nonlinear association of PM_10_ with the predictors (only four predictors i.e. Mg, PO_4_, Ca and Al are shown for brevity). The dashed lines show the confident intervals and the vertical lines on the *x*-axis show the data presence.

Figure 6 shows the nonlinear association between PM_10_ and some of the predictors. Their mutual association varied with the levels of the predictor variables. For example, in the case of Al, there was a positive association between Al and PM_10_; however, when Al level reached about 1.8 µg/m^3^, the nature of association changed from positive to negative, which meant PM_10_ levels decreased with increasing Al. When Al levels reached about 2.8, the association changed again, and the curve became parallel with the *x*-axis (horizontal), meaning PM_10_ levels did not change with further increasing Al levels approximately from 2.8–4.0 µg/m^3^. However, it should be noted that at higher levels of Al, there were fewer data points (shown by the vertical black ticks on the *x*-axis), and therefore the uncertainties levels increased (shown by the dotted lines on both sides of the solid line). Therefore, at higher levels, the association between the two variables became weaker and less reliable. For more detail on the nonlinear association between PM_10_ and the predictors, see Figure 6. The nonlinear relationship between PM_10_ and the predictor variables was probably due to changes in emission sources and meteorological conditions that controlled the levels of PM_10_ and the predictor variables.

### 3.4. Enrichment Factors (EF)

Enrichment factors (EF) were calculated for the TE analysed in this paper (Table 1). Concentrations of TE in PM_10_ and earth-crust are given in Table 1. The ratios of TE to Al in PM_10_ were divided by their ratios in earth-crust to get the values of EF (Table 1). As mentioned above, all TEs having EF higher than 10 (EF > 10), were considered to be predominantly emitted by anthropogenic sources, whereas TE having EF less than 10 (EF < 10) were considered to have originated from the earth-crust. According to the data presented in Table 1, only two TE had EF higher than 10, which were Na and Ca. All the rest of TE had EF less than 10. Therefore, EF analysis showed that most of the TE originated from the earth-crust, except Na and Ca, which were attributed to anthropogenic sources. The shortcoming of the EF analysis here was that the elemental composition of local earth-crust was not available, which might have affected the outcome of the analysis. Khodeir et al. [9] and Nayebare et al. [8] have also used EF in their analysis for sources identification in Jeddah and Makkah, respectively. According to [8], the anthropogenic TE were Cu, Zn, Eu, Cl, Pb, S, Br, and Lu, whereas the TE derived from the earth-crust were Al, Si, Na, Mg, Rb, K, Zr, Ti, Fe, Mn, Sr, Y, Cr, Ga, Ca, Ni, and Ce.

### 3.5. Positive Matrix Factorization (PMF)

**Figure 7:**
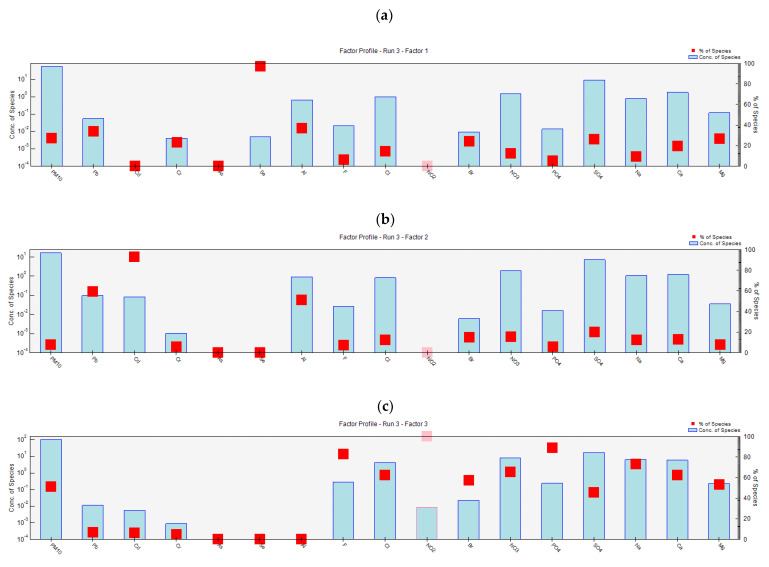

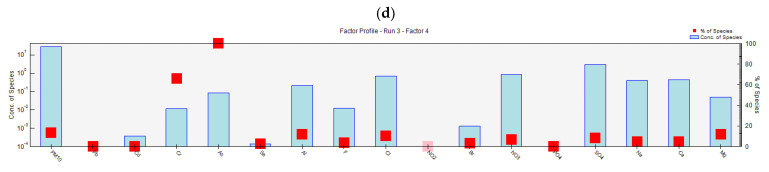
Results of the positive matrix factorization (PMF) run 3 (the convergent run), showing profiles % of species for factor 1 (**a**), factor 2 (**b**), factor 3 (**c**) and factor 4 (**d**).

The outcomes of PMF are presented in Figure 7 and Table 2. Four factors were identified and ascribed as the four major sources of PM_10_ in Makkah. Factor fingerprints, factor profiles, and factor contributions to PM_10_ are presented in Figure 7 and Table 2. Factor 1 (industrial emissions and mineral dust) explained 27.5%, factor 2 (fossil fuel combustion) explained 7.9%, factor 3 (Road traffic) explained 51.0%, and factor 4 (restaurants and dwellings) explained 13.6% of the variations in PM_10_ concentrations (Figure 7). Looking at the fingerprints of different factors, it can be observed that most of the elements contributed to more than one factor, meaning they were emitted by more than one emission source in different proportions, except As and NO_2_, which contributed to only a single factor each, i.e., factor 4 and 3, respectively. Se and Cd contributed to only two factors each, i.e., factors 1 and 4 and 2 and 3, respectively. This showed that different emission sources contributed to the emission of a chemical species in various proportions. Such analysis helped us identify the main sources of emission, as explained below. The four factors of PMF were identified as four emission sources of PM_10_ in Makkah based on the loadings of chemical species in each factor.

Road traffic (exhaust and non-exhaust emission and resuspension of dust): Factor 3 explained the highest proportion (51%) of the PM_10_ concentration. Factor 3 was identified as road traffic emission because the pollutants are related to vehicle exhaust, non-exhaust, or resuspension of dust on roadsides [8]. Factor 3 mainly consisted of NO_2_, F, Cl, Br, PO_4_, NO_3_, SO_4_, Na, Ca, and Mg (Figure 7). Minor contributors were Cd, Cr, and Pb. It was reported that Ca, Mg, Na, and Al were some of the major elements emitted by the vehicle’s tyres [8], whereas Pb and Cd were found in the emission from brake wear [54,55].

Industrial emissions and mineral dust: Factor 1 explained the second largest proportion (27.5%) in PM_10_ concentrations. The chemical species that contributed to factor 1 were Se, Al, Pb, Cr, Mg, Ca, SO_4_, Br, Na, NO_3_, PO_4_, Cr, and F. The three main contributors were Se, Pb, and Al. About 98% of Se falls in factor 1, whereas the loadings of Al and Pb were about 40% and 35%, respectively. Forty percent (40%) of Se is reported to be emitted by anthropogenic sources, including coal combustion, metal smelting, and biomass burning [56]. These sources also emitted Al and Pb. Therefore, Factor 1 was identified as industrial emission. However, Na, Ca, Mg, Al, and PO_4_ are the markers for mineral dust [57]. Part of the mineral dust may also come from construction and demolition activities. Regional transport of marine aerosols from the Red Sea also contributed to the concentrations of Na, Cl, PO_4_, and Mg [8].

Fossil fuel combustion: Factor 2 explained a 7.9% variation in PM_10_ concentrations and consisted of Cd, Pb, Al, SO_4_, F, Cr, Br, NO_3_, Na, Ca, and Mg. A large proportion of factor 2 was made of Cd, Pb, and Al. Airborne Cd is predominantly (85–90%) emitted by anthropogenic sources, including fossil fuel combustion, the smelting and refinement of nonferrous metals, and municipal waste incineration [58]. Naturally, Cd is emitted by volcanic eruptions [58]. In addition to road traffic, Pb is also emitted during the open burning of municipal waste [59]. The presence of Al, F, Na, Ca, and Mg also indicated the effect of windblown dust particles, which is common in Makkah due to high temperature, dry weather, and frequent dust storms.

Restaurants and dwellings: Factor 4 explained 13.6% of the variance in PM_10_ concentrations and showed high loadings of As and Cr. Other elements that made a minor contribution to factor 4 were Al, Mg, Ca, Na, SO_4_, NO_3_, Br, Cr, F, and Se. ‘As’ is naturally emitted by volcanic eruptions, vegetation, and windblown dust, whereas anthropogenic sources of ‘As’ include fuel combustion especially burning of low-grade brown coal and metal smelting [58]. Cr is mainly emitted to the atmosphere by burning coal, oil, and natural gas [60]. In Makkah, coal is mainly burnt in restaurants, whereas natural gas is burnt in restaurants and homes. Combustion of coal and natural gas also contributes to the combustion-related pollutants, which include SO_2_ and NO_x_, which in turn are transformed to NO_3_ and SO_4_ ions in the atmosphere.

According to the enrichment factor (EF) analysis, most of the TEs were originated from the earth’s crust, except Na and Ca. However, according to the PMF model, the dominant emission sources, in addition to windblown dust and resuspension of dust on roadsides, in Makkah were road traffic, various industries, combustion of fossil fuels, and combustion of low-grade coal, oil, and natural gas in restaurants and dwellings. Previously, the authors of [8] identified four main sources of PM_2.5_, namely, vehicular emissions (30.1%), industrial-mixed dust (28.9%), soil/earth crust (24.7%), and fossil fuels/oil combustion (16.3%), which generally were in agreement with the findings of the current study. However, in this study, the contribution of restaurants and dwellings was also identified as a significant source. Makkah, due to the presence of the Holy Mosque, attracts millions of visitors every year. These visitors use the local restaurants for their food that burn coal and natural gas for cooking and thus emit a significant amount of pollutants to the atmosphere. In Makkah, several restaurants on each street burn coals and natural gas, especially in the centre of the city around the Holy Mosque. It is important to mention that large-scale construction-and-demolition activities in Makkah also add to the atmospheric load of particulate matter [53]. Previously, Na and Cl have been identified as the markers of sea spray and marine aerosols; however, in this study, their contribution to each factor is minor, probably indicating that these pollutants are emitted by combustion processes and are generated from the earth-crust.

## 4. Conclusions and Recommendation

In this paper, the main aim was to identify the major emission sources of PM_10_ in Makkah, employing various statistical and machine learning approaches, which included: (a) Descriptive statistics and graphical visualization, (b) Correlation analysis, (c) Enrichment factor, (d) Generalised additive model, and (e) Positive matrix factorization. Makkah is one of the densely populated cities in Saudi Arabia, where atmospheric particulate matter is the pollutant of concern. PM_10_ concentrations exceeded WHO (15 µg/m^3^), USEPA (50 µg/m^3^), and the Saudi Arabia national (80 µg/m^3^) annual air quality standards. For the first time, a GAM model was developed using PM_10_ as a response variable and ions and TEs as predictors. GAM analysed the nonlinear association between PM_10_ and its predictors, which varied at different levels of the predictors, and showed that these predictors were able to explain about 88% variation in PM_10_.

PMF identified four main emission sources of PM_10_ in Makkah:

1. Road traffic including both exhaust and non-exhaust emission, and resuspension of dust (explained 51% variance in PM_10_);
2. Industrial emissions and mineral dust (explained 27.5% variance);
3. Restaurants and dwellings (explained 13.6% variance); and
4. Other fossil fuel combustion (explained 7.9%).

In addition to the natural sources of PM_10_ such as windblown dust, several anthropogenic sources were identified that contributed significantly to PM_10_ concentrations. Although it is not feasible to control emissions from natural sources, government policy can be implemented to control emissions from anthropogenic sources, including emissions from road traffic, industries, and restaurants and dwellings. It is interesting to see that burning coals and natural gas in restaurants, hotels, and dwellings were identified as a significant source of particulate matter. However, like other urban areas, in Makkah, the main emission source identified was road traffics, which emit exhaust and non-exhaust emissions and cause resuspension of dust particles on roadsides. The main weakness of the study is that a limited number of ions and TEs were analysed. However, GAM analysis showed that these species explained nearly 90% variations in PM_10_. It is recommended that a detailed emission inventory be compiled in Makkah characterising road traffic flow, fleet composition, point emissions (e.g., industries, power plants), and area emissions (e.g., emission from restaurants, hotels, and dwellings). Furthermore, the impacts of air pollution should be analysed, including its impact on health, economy, and sustainability.

To manage air quality and cut the emission of pollutants from various sources, the following actions are recommended in Makkah:

1. Road traffic is the main emission source of gaseous and particle pollution in Makkah. Therefore, it is important to effectively manage traffic flow to avoid congestion during the peak hours, particularly during the months of the Hajj and Ramadhan [61]. Also, the use of public transport should be encouraged;
2. Further intervention for reducing air pollution in Makkah may include banning old polluting vehicles and retrofitting them with new technology [61];
3. More electric vehicles should be included in the fleet and better charging facilities should be provided [62];
4. To improve driving behaviour, further training should be provided to discourage abrupt acceleration, deceleration and idling [61]. Idling is a serious issue due to extremely high temperatures. Drivers keep their vehicle’s engines on during summer to keep the interior of the vehicle cold using the vehicle’s air conditioners. Otherwise, within a few minutes, the vehicle becomes unbearably hot. Environmentally friendly vehicles and technologies must be used in such situations;
5. As previously reported by several authors [63], trees not only control air pollution but also help moderate air temperature. Therefore, more trees should be grown in the City of Makkah, particularly on roadsides;
6. Large-scale construction and demolition activities increase the loading of atmospheric dust, which could be reduced by an effective water spray programme [64].

## Figures and Tables

**Table 1:** Enrichment factors (EF) of various chemical species.

Element	Earth-Crust Cx	PM_10_ Cx	(Cx/C_Al_) PM_10_	(Cx/C_Al_) Earth-Crust	EF
Al	8.230	1.784	1.000	1.000	1.000
Pb	12.500	0.161	0.090	1.519	0.059
Cd	0.200	0.085	0.048	0.024	1.961
Cr	100.000	0.017	0.010	12.151	0.001
As	1.800	0.085	0.048	0.219	0.218
Se	0.050	0.004	0.002	0.006	0.369
F	525.000	0.330	0.185	63.791	0.003
Cl	130.000	6.837	3.832	15.796	0.243
Br	2.500	0.042	0.024	0.304	0.078
Na	2.360	8.596	4.818	0.287	16.803
Ca	4.150	9.341	5.236	0.504	10.384
Mg	2.330	0.444	0.249	0.283	0.879

Note: The earth-crust concentrations were taken from [47]. Cx is the concentration of an element compared to ‘Al’ (C_Al_).

**Table 2:** Results of the positive matrix factorization run 3 (the convergent run), showing profile % of species, % of factor total and concentrations of species in each factor (factor 1 to factor 4).

	Profile (% of Species)				Profile (% of Factor Total)				Profile (Concentration of Species)			
	Factor 1	Factor 2	Factor 3	Factor 4	Factor 1	Factor 2	Factor 3	Factor 4	Factor 1	Factor 2	Factor 3	Factor 4
PM_10_	27.48	7.87	51.01	13.64	78.59	54.56	71.52	82.83	56.66	16.23	105.16	28.11
Pb	34.04	59.22	6.74	<0.01	0.08	0.33	0.01	<0.01	0.06	0.10	0.01	<0.01
Cd	<0.01	93.19	6.39	0.42	<0.01	0.28	<0.01	<0.01	<0.01	0.08	0.01	<0.01
Cr	23.23	5.81	4.88	66.08	0.01	<0.01	<0.01	0.03	<0.01	<0.01	<0.01	0.01
As	<0.00	<0.01	<0.01	100.00	<0.01	<0.01	<0.01	0.25	<0.01	<0.01	<0.01	0.08
Se	97.28	<0.01	<0.01	2.72	<0.01	<0.01	<0.01	<0.01	<0.01	<0.01	<0.01	<0.01
Al	36.74	51.03	<0.01	12.22	0.91	3.06	0.00	0.64	0.66	0.91	<0.01	0.22
F	6.28	7.49	82.64	3.59	0.03	0.09	0.19	0.04	0.02	0.03	0.28	0.01
Cl	14.51	12.38	62.54	10.56	1.34	2.78	2.84	2.08	0.97	0.83	4.17	0.70
NO_2_	<0.01	<0.01	100.00	<0.01	<0.01	<0.01	0.01	<0.01	<0.01	<0.01	0.01	<0.01
Br	24.14	15.25	57.36	3.25	0.01	0.02	0.02	<0.01	0.01	0.01	0.02	<0.01
NO_3_	12.25	15.71	65.40	6.63	2.15	6.67	5.62	2.47	1.55	1.98	8.26	0.84
PO_4_	5.16	5.88	88.96	<0.01	0.02	0.05	0.16	<0.01	0.01	0.02	0.24	<0.01
SO_4_	26.01	19.96	45.61	8.41	13.08	24.32	11.24	8.99	9.43	7.24	16.53	3.05
Na	9.19	12.61	73.42	4.78	1.09	3.63	4.27	1.21	0.79	1.08	6.29	0.41
Ca	19.59	13.10	62.53	4.78	2.53	4.10	3.96	1.31	1.83	1.22	5.83	0.45
Mg	27.02	8.09	53.11	11.77	0.16	0.12	0.16	0.15	0.12	0.04	0.23	0.05

## Data Availability

Not applicable.
